# Incidence rate of recurrent cardiovascular events in patients with radiographic axial spondyloarthritis and the effect of tumor necrosis factor inhibitors

**DOI:** 10.1186/s13075-024-03405-7

**Published:** 2024-10-04

**Authors:** Oh Chan Kwon, Hye Sun Lee, So Young Jeon, Min-Chan Park

**Affiliations:** 1 Division of Rheumatology, Department of Internal Medicine, Yonsei University College of Medicine, Seoul, South Korea; 2 Biostatistics Collaboration Unit, Yonsei University College of Medicine, Seoul, South Korea; 3 Gangnam Severance Hospital, Yonsei University College of Medicine, 211 Eonjuro, Gangnam-gu, Seoul, 06273 South Korea

**Keywords:** Radiographic axial spondyloarthritis, Tumor necrosis factor inhibitors, Cardiovascular, Recur, Risk

## Abstract

**Background:**

Patients with radiographic axial spondyloarthritis (r-axSpA) are at increased risk of incident cardiovascular events. Tumor necrosis factor inhibitors (TNFi) have shown a protective effect against incident cardiovacular events. However, the incidence of recurrent cardiovascular events in patients with r-axSpA with a history of cardiovascular events, and the effect of TNFi on recurrent cardiovascular events remain unclear. We aimed to assess the incidence rate of recurrent cardiovascular events in patients with r-axSpA with a history of cardiovascular events and evaluate the effect of TNFi on the risk of recurrent cardiovascular events.

**Methods:**

This nationwide cohort study used data from the Korean National Claims Database. Data of patients with r-axSpA who had a history of cardiovascular events after being diagnosed with r-axSpA were extracted from the database. The outcome of interest was the recurrence of cardiovascular events (myocardial infarction or stroke). Patients were followed from the index date (date of the first cardiovascular event) to the date of cardiovascular event recurrence, the last date with claims data, or December 31, 2021, whichever occured first. The incidence rate of recurrent cardiovascular events was calculated. An inverse probability weighted Cox model was used to assess the effect of TNFi exposure on the risk of recurrent cardiovascular events.

**Results:**

This study included 413 patients (TNFi non-exposure, *n* = 338; TNFi exposure, *n* = 75). The incidence rate of recurrent cardiovascular events was 32 (95% confidence interval [CI] 22–42) per 1,000 person-years (TNFi non-exposure, 36 [95% CI 24–48] per 1,000 person-years; TNFi exposure, 19 [95% CI 2–35] per 1,000 person-years). In the inverse probability weighted Cox model, TNFi exposure was significantly associated with a lower risk of recurrent cardiovascular events (hazard ratio 0.33, 95% CI 0.12–0.94).

**Conclusions:**

The incidence rate of recurrent cardiovascular events in patients with r-axSpA is substantial. TNFi exposure was associated with a lower risk of recurrent cardiovascular events.

## Background

Individuals with a history of cardiovascular events have a high risk of recurrent cardiovascular events [1]. Therefore, secondary prevention of cardiovascular events in patients with a history of cardiovascular events is important [2]. Antiplatelet agents, statins, and colchicine are medications that are used for the secondary prevention of cardiovascular events [3–5]. In addition, lifestyle modification is also important for secondary prevention of cardiovascular events [6]. Patients with radiographic axial spondyloarthritis (r-axSpA) have a 1.4-fold higher risk of cardiovascular events than the general population [7]. Moreover, considering that cardiovascular events are associated with a two-fold higher risk of mortality in patients with r-axSpA, cardiovascular events are important comorbidities that should be considered when treating patients with r-axSpA [8]. The higher risk of cardiovascular events in r-axSpA is attributed to the inflammatory nature of the disease [9]. Independent of traditional cardiovascular risk factors, inflammation accelerates atherosclerosis and increases the risk of cardiovascular events [9–11]. Studies have shown that the use of tumor necrosis factor inhibitors (TNFi), which are biological disease-modifying anti-rheumatic drugs (bDMARDs) that are effective in controlling inflammation in r-axSpA, is associated with a reduced risk of cardiovascular events [12, 13].

Although it is well known that patients with r-axSpA have a higher incidence rate of cardiovascular events than the general population [7, 14], the incidence rate of recurrent cardiovascular events in patients with r-axSpA is poorly studied. Moreover, although the beneficial effects of TNFi on the risk of incident cardiovascular events in patients with r-axSpA have been reported [12, 13], it is unclear whether TNFi are also effective in reducing the risk of recurrent cardiovascular events (i.e., in a setting where medications such as antiplatelet agents and statins are being used for secondary prevention).

This study used a nationwide database to assess the incidence rate of recurrent cardiovascular events in patients with r-axSpA and evaluate the effect of TNFi on the risk of recurrent cardiovascular events.

## Patients and methods

### Data source

Data were extracted from the Health Insurance Review and Assessment Service (HIRA) database, a nationwide database that includes approximately 97% of the entire population of South Korea. Comprehensive data, including demographics, disease diagnosis based on the International Classification of Diseases, Tenth Revision (ICD-10) code and rare intractable disease (RID) code, medications, and medical procedures, are included in the HIRA database [15]. The RID code is registered after a thorough review by the National Health Insurance (NHI) and the corresponding healthcare institution to ensure compliance with the uniform diagnostic criteria provided by the NHI [16].

### Study cohort

**Fig. 1:**
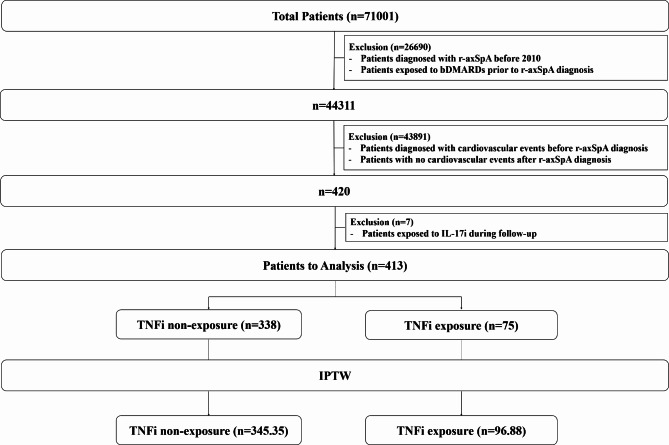
Flowchart of the study. r-axSpA, radiographic axial spondyloarthritis; bDMARDs, biological disease-modifying antirheumatic drugs; IL-17i, interleukin-17 A inhibitors; TNFi, tumor necrosis factor inhibitors

All patients with r-axSpA were initially screened from the database. r-axSpA was defined as ICD-10 code M45 with RID code V140 [16]. Patients with the following criteria were excluded: (i) diagnosis of r-axSpA before 2010, (ii) any bDMARDs exposure prior to the diagnosis of r-axSpA, (iii) cardiovascular events (myocardial infarction [MI] or stroke) prior to the diagnosis of r-axSpA (as patients with cardiovascular events prior to r-axSpA diagnosis might have different risk of recurrent cardiovascular events before and after they are diagnosed with r-axSpA, patients with cardiovascular events prior to the r-axSpA dianosis were excluded to avoid the influence of r-axSpA itself), (iv) no cardiovascular events after the diagnosis of r-axSpA, and (v) exposure to interleukin-17 A inhibitors (IL-17i) during follow-up. As a result, 413 patients with r-axSpA who were diagnosed with cardiovascular events after r-axSpA diagnosis were included in the analysis (Fig. 1). The index date was defined as the date of the first diagnosis of cardiovascular events. Patients were followed from the index date to the date of cardiovascular event recurrence, the last date with claims data, or December 31, 2021, whichever occured first.

This study was approved by the Institutional Review Board (IRB) of Gangnam Severance Hospital (IRB No: 3-2023-0271). The HIRA database provides fully anonymized data, and therefore, the requirement for informed consent was waived.

### Exposure

Exposure to TNFi was assessed as a binary variable (ever used during follow-up: yes or no). According to the national reimbursement policy of South Korea, TNFi can be initiated in patients with active disease despite treatment with non-steroidal anti-inflammatory drugs (NSAIDs) with or without conventional synthetic DMARDs for at least 3 months.

### Covariates and outcome

Age, sex, disease duration of r-axSpA, and the presence of hypertension, type 2 diabetes, dyslipidemia, and chronic kidney disease (CKD) on the index date were included as covariates. Hypertension was defined as ICD-10 codes I10‒I13 and I15 with prescriptions for antihypertensive agents; type 2 diabetes was defined as ICD-10 codes E11–14 and at least one annual claim of a prescription of antidiabetic agents; dyslipidemia was defined as ICD-10 code E78 with prescriptions for lipid-lowering agents; and CKD was defined as ICD-10 code N18 or N19 [17, 18]. Medications (methotrexate, sulfasalazine, glucocorticoids, non-selective NSAIDs, selective cyclooxygenase-2 [COX-2] inhibitors, antiplatelet agents, anticoagulants, statins, angiotensin-converting enzyme inhibitors or angiotensin II receptor blockers, beta-blockers, sodium-glucose cotransporter-2 inhibitors, and glucagon-like peptide-1 receptor agonists) used during the follow-up were also included as covariates.

The outcome of this study was recurrent cardiovascular events (MI or stroke). MI was defined as ICD-10 code I21 or I22 during hospitalization; stroke was defined as ICD-10 code I63 or I64 during hospitalization with claims for brain magnetic resonance imaging or computed tomography [19]. Any MI or stroke event that occurred at least 28 days after the first event was considered a recurrence [20].

### Statistical analyses

Patients were categorized into TNFi non-exposure and TNFi exposure groups. Characteristics are summarized as mean (± standard deviation) or numbers (%) for continuous or categorical variables, respectively. Student’s t-test was used for comparing continuous variables, and the χ2 test or Fisher’s exact test was used for comparing categorical variables between the two groups (TNFi non-exposure vs. TNFi exposure). The number of outcomes (%) and crude incidence rates were calculated for the TNFi non-exposure and exposure groups, respectively.

To mitigate confounding by indication, we used inverse probability of treatment weighting (IPTW) based on the propensity score. The propensity score for TNFi exposure was estimated using a multivariable logistic regression model that included age, sex, disease duration, hypertension, type 2 diabetes, dyslipidemia, and CKD. Patients who were not exposed to TNFi were assigned a weight of 1/(1–propensity score) and those who were exposed to TNFi were assigned a weight of 1/propensity score. After IPTW, continuous variables were expressed as the weighted mean (standard error), and categorical variables were expressed as weighted numbers (%). An inverse probability weighted Cox regression analysis was performed to assess the hazard ratio (HR) and 95% confidence interval (CI) of recurrent cardiovascular events according to TNFi exposure. A univariable model was used, followed by a multivariable model adjusted for medications (use of methotrexate, sulfasalazine, glucocorticoids, non-selective NSAIDs, selective COX-2 inhibitors, antiplatelet agents, anticoagulants, statins, angiotensin-converting enzyme inhibitors or angiotensin II receptor blockers, beta-blockers, sodium-glucose cotransporter-2 inhibitors, and glucagon-like peptide-1 receptor agonists). All reported p-values are two-sided, and statistical significance was set at *p* < 0.05. Statistical analyses were performed using SAS version 9.4 (SAS Institute, Cary, NC, USA).

## Results

### Patient characteristics

Of the 413 patients who were diagnosed with cardiovascular events after r-axSpA diagnosis, 338 and 75 were unexposed and exposed to TNFi, respectively, during follow-up. In the TNFi exposure group, the mean duration of TNFi exposure was 2.17 ± 2.12 years. A comparison of the characteristics of the two groups is shown in Table 1. Patients in the non-exposure group were older (60.7 ± 13.3 years vs. 51.9 ± 10.9 years, *p* < 0.001), less commonly men (74.9% vs. 92.0%, *p* = 0.001), had higher hypertension (64.8% vs. 42.7%, *p* < 0.001) and type 2 diabetes (28.1% vs. 16.0%, *p* = 0.030) rates, and less commonly took methotrexate (11.2% vs. 28.0%, *p* < 0.001), sulfasalazine (38.2% vs. 73.3%, *p* < 0.001), and selective COX-2 inhibitors (59.8% vs. 72.0%, *p* = 0.048) than those in the exposure group.

**Table 1:** Characteristics of the study population before IPTW

Variable	TNFi non-exposure (*n* = 338)	TNFi exposure (*n* = 75)	*p* value
Age, years, mean ± SD	60.7 ± 13.3	51.9 ± 10.9	< 0.001
Male, n (%)	253 (74.9)	69 (92.0)	0.001
Disease duration, years, mean ± SD	3.2 ± 2.8	3.2 ± 2.9	0.918
Comorbidities			
Hypertension, n (%)	219 (64.8)	32 (42.7)	< 0.001
Type 2 diabetes, n (%)	95 (28.1)	12 (16.0)	0.030
Dyslipidemia, n (%)	149 (44.1)	33 (44.0)	0.990
Chronic kidney disease, n (%)	23 (6.8)	1 (1.3)	0.097
Medications			
Methotrexate, n (%)	38 (11.2)	21 (28.0)	< 0.001
Sulfasalazine, n (%)	129 (38.2)	55 (73.3)	< 0.001
Glucocorticoids, n (%)	305 (90.2)	67 (89.3)	0.813
Non-selective NSAIDs, n (%)	319 (94.4)	71 (94.7)	> 0.999
Selective COX-2 inhibitors, n (%)	202 (59.8)	54 (72.0)	0.048
Antiplatelet agents, n (%)	325 (96.2)	75 (100.0)	0.138
Anticoagulants, n (%)	61 (18.0)	7 (9.3)	0.066
Statins, n (%)	315 (93.2)	71 (94.7)	0.799
ACE inhibitors or ARBs, n (%)	259 (76.6)	58 (77.3)	0.896
Beta-blockers, n (%)	219 (64.8)	44 (58.7)	0.318
SGLT2 inhibitors, n (%)	37 (10.9)	11 (14.7)	0.363
GLP1 receptor agonists, n (%)	3 (0.9)	0 (0.0)	> 0.999

ACE, angiotensin-converting enzyme; ARBs, angiotensin II receptor blockers; COX-2, cyclooxygenase-2; GLP1, glucagon-like peptide-1; IPTW, inverse probability of treatment weighting; NSAIDs, non-steroidal anti-inflammatory drugs; SD, standard deviation; SGLT2, sodium-glucose cotransporter-2; TNFi, tumor necrosis factor inhibitors

### Incidence rate of recurrent cardiovascular events

In the total study population, 38 cardiovascular events (MI, 33; stroke, 5) recurred during 1187 person-years of follow-up, accounting for an incidence rate of 32 (95% CI 22–42) per 1,000 person-years. In the non-exposure group, 33 cardiovascular events recurred during 917.32 person-years of follow-up, accounting for an incidence rate of 36 (95% CI 24–48) per 1,000 person-years. In the exposure group, five cardiovascular events recurred during 270 person-years of follow-up, accounting for an incidence rate of 19 (95% CI 2–35) per 1,000 person-years (Table 2).

**Table 2:** Crude incidence rate of recurrence

	Events	Time to events, years, mean ± SD	Time at risk, years, mean ± SD	Total person-years	IR per 1,000 person-years (95% CI)
Total study population	38	1.59 ± 2.11	2.88 ± 2.57	1187	32 (22–42)
TNFi non-exposure	33	1.54 ± 2.20	2.71 ± 2.46	917	36 (24–48)
TNFi exposure	5	1.93 ± 1.46	3.60 ± 2.92	270	19 (2–35)

CI, confidence interval; IR, incidence rate; SD, standard deviation; TNFi, tumor necrosis factor inhibitors

### After IPTW

A comparison of the characteristics of the two groups after IPTW is presented in Table 3. There were no significant differences in age, sex, disease duration, and presence of comorbidities between the two groups. With regard to medications, methotrexate (11.14% vs. 29.87%, *p* < 0.001), sulfasalazine (40.54% vs. 75.26%, *p* < 0.001), and selective COX-2 inhibitors (58.34% vs. 74.55%, *p* = 0.017) were less commonly used in the non-exposure group than in the exposure group. Compared with the non-exposure group, the exposure group showed numerically fewer recurrent cardiovascular events (9.72% vs. 4.69%, *p* = 0.132), although statistical significance was not reached.

**Table 3:** Characteristics of the study population after IPTW

Variable	TNFi non-exposure (*n* = 345.35)	TNFi exposure (*n* = 96.88)	*p* value
Age, years, weighted mean (SE)	59.01 (0.78)	57.01 (1.76)	0.299
Male, weighted n (%)	269.63 (78.03)	78.7 (81.23)	0.697
Disease duration, years, weighted mean (SE)	3.21 (0.16)	3.09 (0.37)	0.758
Comorbidities			
Hypertension, weighted n (%)	208.97 (60.48)	53.78 (55.51)	0.493
Type 2 diabetes, weighted n (%)	88.99 (25.76)	19.48 (20.11)	0.378
Dyslipidemia, weighted n (%)	146.79 (42.48)	47.68 (49.22)	0.367
Chronic kidney disease, weighted n (%)	20.01 (5.79)	3.97 (4.09)	0.723
Medications			
Methotrexate, weighted n (%)	38.48 (11.14)	28.94 (29.87)	< 0.001
Sulfasalazine, weighted n (%)	140.07 (40.54)	72.91 (75.26)	< 0.001
Glucocorticoids, weighted n (%)	311.36 (90.11)	86.74 (89.53)	0.887
Non-selective NSAIDs, weighted n (%)	327.25 (94.71)	92.76 (95.74)	0.698
Selective COX-2 inhibitors, weighted n (%)	201.59 (58.34)	72.22 (74.55)	0.017
Antiplatelet agents, weighted n (%)	330.99 (95.79)	96.88 (100.00)	NA
Anticoagulants, weighted n (%)	59.53 (17.23)	13.15 (13.58)	0.566
Statins, weighted n (%)	322.22 (93.25)	92.80 (95.79)	0.392
ACE inhibitors or ARBs, weighted n (%)	260.78 (75.47)	77.90 (80.40)	0.401
Beta-blockers, weighted n (%)	221.52 (64.11)	58.14 (60.01)	0.565
SGLT2 inhibitors, weighted n (%)	37.35 (10.81)	11.24 (11.60)	0.839
GLP1 receptor agonists, weighted n (%)	3.09 (0.89)	0.00 (0.00)	NA
Recurrence, weighted n (%)	33.60 (9.72)	4.54 (4.69)	0.132

ACE, angiotensin-converting enzyme; ARBs, angiotensin II receptor blockers; COX-2, cyclooxygenase-2; GLP1, glucagon-like peptide-1; IPTW, inverse probability of treatment weighting; NSAIDs, non-steroidal anti-inflammatory drugs; SE, standard error; SGLT2, sodium-glucose cotransporter-2; TNFi, tumor necrosis factor inhibitors

### Risk of recurrent cardiovascular events according to TNFi exposure

In the univariable model of inverse probability weighted Cox regression analysis, TNFi exposure showed a trend toward a lower risk of recurrent cardiovascular events than TNFi non-exposure (HR 0.39, 95% CI 0.15–1.05, *p* = 0.063). After adjusting for medications in the multivariable model, TNFi exposure was significantly associated with a lower risk of recurrent cardiovascular events than TNFi non-exposure (HR 0.33, 95% CI 0.12–0.94, *p* = 0.038) (Table 4).

**Table 4:** Inverse probability-weighted Cox models

	Univariable model		Multivariable model*	
	HR (95% CI)	p value	HR (95% CI)	p value
TNFi non-exposure	1.00		1.00	
TNFi exposure	0.39 (0.15–1.05)	0.063	0.33 (0.12–0.94)	0.038

*Adjusted for use of methotrexate, sulfasalazine, glucocorticoids, non-selective non-steroidal anti-inflammatory drugs, selective cyclooxygenase-2 inhibitors, antiplatelet agents, anticoagulants, statins, angiotensin-converting enzyme inhibitors or angiotensin II receptor blockers, beta-blockers, sodium-glucose cotransporter-2 inhibitors, and glucagon-like peptide-1 receptor agonists

CI, confidence interval; HR, hazard ratio; TNFi, tumor necrosis factor inhibitors

## Discussion

This nationwide cohort study revealed that the incidence rate of recurrent cardiovascular events in patients with r-axSpA with a prior history of cardiovascular events was 32.00 per 1,000 person-years. Furthermore, TNFi exposure was associated with a lower risk of recurrent cardiovascular events than TNFi non-exposure. Considering that cardiovascular events are important comorbidities that contribute to a higher risk of mortality in patients with r-axSpA [8], the data presented in our study hold clinical importance.

Previous observational studies and meta-analyses have reported the incidence rate of cardiovascular events (defined as MI or stroke) in patients with r-axSpA, which ranges from 3.6 to 7.0 per 1,000 person-years [7, 13, 21]. However, the incidence rate of recurrent cardiovascular events has not been reported. The present study found that the incidence rate of recurrent cardiovascular events in patients with r-axSpA was 32 per 1,000 person-years, which is markedly higher than the incidence rate of first cardiovascular events reported previously. Notably, the incidence rate of recurrent cardiovascular events in our study population was similar to that observed in patients with type 2 diabetes (33.0 per 1,000 person-years) [20], a well-known population with a high risk of cardiovascular events [22, 23]. The high incidence rate of recurrent cardiovascular events in patients with r-axSpA with prior cardiovascular events reflects the importance of secondary prevention in these patients.

Although previous studies have reported the beneficial effect of TNFi on the risk of cardiovascular events in patients with r-axSpA [12, 13], these results cannot be generalized to the setting of recurrent cardiovascular events because patients are taking medications for secondary prevention in this setting. As our study population comprised patients with r-axSpA and a prior history of cardiovascular events, the majority of the patients were taking antiplatelet agents (400 of 413 patients, 96.9%) and statins (386 of 413 patients, 93.5%) for the secondary prevention of cardiovascular events. In the multivariable model of inverse probability-weighted Cox regression analysis, TNFi exposure was associated with a significantly lower risk (HR 0.33, 95% CI 0.12–0.94, *p* = 0.038) of recurrent cardiovascular events in patients with r-axSpA with a prior history of cardiovascular events. This suggests that TNFi could be beneficial in reducing the risk of recurrent cardiovascular events, even in patients taking antiplatelet agents and statins for secondary prevention.

Inflammatory bowel disease (IBD) is another immune-mediated inflammatory disease in which TNFi are used for treatment [24]. Similar to our finding, a recent French nationwide study reported that TNFi exposure was associated with a lower risk (HR 0.75, 95% CI 0.63–0.90) of recurrent cardiovascular events compared with TNFi non-exposure in patients with IBD [25]. In contrast, a meta-analysis of randomized controlled trials testing the effect of DMARDs on cardiovascular events in patients with rheumatoid arthritis, psoriasis, heart failure, and MI showed that none of the DMARD subclasses (TNFi, janus kinase inhibitors, and interleukin inhibitors) had effect on cardiovascular events [26]. However, data on patients with r-axSpA are lacking. To our knowledge, this study is the first to evaluate and demonstrate that TNFi is beneficial in reducing the risk of recurrent cardiovascular events in patients with r-axSpA.

TNF-α is involved in the acceleration of atherosclerosis and rupture of atherosclerotic plaques [27, 28]. Moreover, a Mendelian randomization study revealed evidence of a causal association between TNF-α levels and cardiovascular events (coronary artery disease and stroke) [29]. These data indicate that TNFi is beneficial in reducing the risk of the first occurrence of cardiovascular events. Regarding the setting of recurrent cardiovascular events, studies have shown that TNF-α is upregulated in the myocardium in response to myocardial ischemia and reperfusion [30–32]. Moreover, elevated plasma levels of TNF-α post-MI are associated with an increased risk of recurrent cardiovascular events [33]. These data suggest that TNFi might be protective not only for the first occurrence of cardiovascular events but also for recurrent cardiovascular events. Our data add to previous knowledge by providing more direct evidence that TNFi could reduce the risk of recurrent cardiovascular events.

In IPTW, medications were not included as covariates in calculating the propensity score for TNFi exposure because the propensity score was not calculable when medications were included. As a result, the use of some medications (methotrexate, sulfasalazine, and selective COX-2 inhibitors) remained different between the two groups after IPTW. To address confounding by medications, we used a multivariable model to adjust for medications. Covariate adjustment using a multivariable model is one of the methods used for adjusting for confounders [34].

The limitations of this study include: First, although several traditional cardiovascular risk factors, such as hypertension, type 2 diabetes, and dyslipidemia, were well balanced between the two groups after IPTW, we lacked data on other risk factors, such as obesity, smoking and lifestyle, and were unable to adjust for these covariates. Thus, residual confounding may exist. Second, the number of patients in the TNFi exposure group was insufficient to analyze each TNFi (adalimumab, etanercept, golimumab, and infliximab) separately. Whether monoclonal antibody TNFi and receptor fusion protein TNFi have differential effects on the risk of recurrent cardiovascular events requires further study. Furthermore, the number of events, especially the number of recurrent stroke, was relatively small and it was not feasible to analyze MI and stroke separately. In addition, the number of patients who were exposed to IL-17i during follow-up was notably small (*n* = 7) and was excluded from the analysis. Further studies assessing the effect of IL-17i on the risk of recurrent cardiovascular events in patients with r-axSpA are also needed. Third, only Koreans were included in our study, and generalizability to other ethnic populations is unclear. Fourth, this was a open-label, and non-randomized study. Further double-blinded randomized studies involving different ethnic populations would be beneficial.

## Conclusions

In conclusion, we observed a substantial incidence of recurrent cardiovascular events in patients with r-axSpA with a prior history of cardiovascular events, with an incidence rate of 32 per 1,000 person-years. TNFi exposure was associated with a lower risk of recurrent cardiovascular events. This study provides comprehensive data on recurrent cardiovascular events in patients with r-axSpA, providing crucial information for physicians treating patients with r-axSpA and a history of cardiovascular events.

## Data Availability

The data underlying this article are available in the article.

## Abbreviations

r-axSpA: Radiographic axial spondyloarthritis
TNFi: Tumor necrosis factor inhibitors
bDMARDs: biological disease-modifying anti-rheumatic drugs
HIRA: Health Insurance Review and Assessment Service
ICD-10: International Classification of Diseases, Tenth Revision
RID: Rare intractable disease
NHI: National Health Insurance
MI: Myocardial infarction
IL-17i: Interleukin-17 A inhibitors
IRB: Institutional Review Board
NSAIDs: Non-steroidal anti-inflammatory drugs
CKD: Chronic kidney disease
COX-2: Cyclooxygenase-2
IPTW: Inverse probability of treatment weighting
HR: Hazard ratio
CI: Confidence interval
IBD: Inflammatory bowel disease
